# Hepatitis a Vaccine as Opportunity of Primary Prevention for Food Handlers: A Narrative Review

**DOI:** 10.3390/vaccines11071271

**Published:** 2023-07-21

**Authors:** Alessandra Fallucca, Vincenzo Restivo, Maria Chiara Sgariglia, Marco Roveta, Cecilia Trucchi

**Affiliations:** 1 Department of Health Promotion, Maternal and Infant Care, Internal Medicine and Medical Specialties (PROMISE) “G. D’Alessandro”, University of Palermo, 90127 Palermo, Italy; 2 Department of Health Sciences, University of Genoa, 16132 Genoa, Italy; 3 Food Hygiene and Nutrition Service, Department of Prevention, Local Health Unit 3, 16142 Genoa, Italy

**Keywords:** HAV, prevention, vaccine, epidemiology, outbreak, immunogenicity, foodborne viruses, food handlers, food safety

## Abstract

The hepatitis A virus (HAV) is still a leading cause of viral hepatitis worldwide. After a long incubation period, the clinical manifestations range from asymptomatic infection to acute liver failure. The severity of the disease increases with age and pre-existing liver disease. The transmission is mainly via person-to-person contact or ingestion of contaminated food or water. Food contamination can occur at any step of the food chain, especially when infected people handle not-heated or otherwise-treated food. HAV is endemic in low-income countries because of poor sanitary and sociodemographic conditions. The populations of developed countries are highly susceptible, and large outbreaks occur when HAV is introduced from endemic countries due to globalization, travel, and movement of foodstuffs. HAV prevention includes hygiene practices, immunoglobulins, and vaccination. Safe and effective inactivated and live attenuated vaccines are available and provide long-term protection. The vaccine targets are children and subjects at increased risk of HAV exposure or serious clinical outcomes. This review discusses the critical role of food handlers in the spread of HAV and the opportunity for food industry employers to consider food handler immunization a tool to manage both food safety in compliance with HACCP principles and food operators’ biologic risk.

## 1. Introduction

Hepatitis A is a vaccine-preventable disease caused by highly contagious hepatitis A virus (HAV) infection, primarily transmitted through close contact with an infected person or via faecal contamination of food, fomites, or water [1,2,3,4,5,6,7].

HAV is a non-enveloped virus belonging to the *Hepatovirus* genus within the *Picornaviridae* family; its genome consists of a single positive-strand RNA molecule [6,8,9,10,11]. Considering the heterogenicity of the genome, several genotypes and sub-genotypes have been described. Conversely, only a single serotype is known, because heterogeneity has a limited effect on antigenic diversity [10,12,13,14,15,16,17].

Of note, the globalized food chain can represent a relevant contributor to the spread of different HAV variants [18]. In order to conduct epidemiologic surveillance, to track the microbial source, and identify pathogens involved in foodborne outbreaks, genomic epidemiology of HAV is of increasing relevance [19]. Nevertheless, the titres of HAV in humans and food samples are usually low, making the identification and sequencing of the virus challenging [20].

Despite the abovementioned criticisms in epidemiological estimates, the contamination of food with HAV was found to account for 2–7% of all HAV outbreaks, often involving large numbers of affected persons [21].

Many different food items, such as fresh produce (e.g., berry fruits and leafy green vegetables), shellfish (e.g., bivalve mollusks), and crop products, have been associated with HAV outbreaks [22,23,24,25,26,27,28,29,30,31]. Viral contamination may be acquired during cultivation or direct contact with faecal-polluted water, contaminated tools, or faecally contaminated hands of infected food handlers during harvesting, preparation, or distribution [32,33,34,35].

The highest infectivity due to faecal virus shedding occurs from approximately two weeks prior to the onset of jaundice to one week after onset [7,36,37,38]. After the incubation period, lasting usually about 28 days (range 15–50 days), clinical illness typically resolves within two months. Nevertheless, HAV disease presentation is extremely variable depending on the age at which the infection is contracted, with a range from the absence of signs and symptoms to acute liver failure, which is often fatal. About 10–15% of patients develop prolonged or relapsing symptoms for up 6 months [7,9,39,40,41,42,43,44]. Unlike hepatitis B and C, HAV cannot cause chronic liver infection. However, in patients with chronic liver disorders, acute HAV infection can induce liver failure [45].

There is no specific therapy for HAV acute hepatitis. Therefore, symptomatic treatment is indicated [43].

According to global estimates, approximately 600 million cases of foodborne illness related to 31 pathogens are reported annually [46,47]. In 2019, HAV infection caused 159 million cases, resulting in 39,000 deaths and 2.3 disability-adjusted life years (DALYs) [48,49]. However, this figure seems to be underestimated, and the infection rate is probably much higher, making hepatitis A a global public health concern [9,50]. The seroprevalence of HAV infection varies in different regions of the world, being strongly correlated with socioeconomic factors, access to clean water, and proper sanitation [51,52,53]. Improvements in socioeconomic condition, sanitation, and personal hygiene and vaccination play a fundamental role in the prevention of HAV infection [54,55,56].

Effective and safe hepatitis A vaccines have been available since the early 1990s and have proven to be safe and highly immunogenic, resulting in long-lasting immunization [57,58,59].

Some countries and institutions have recommended targeted immunizations of groups at increased risk of HAV exposure or serious clinical outcomes after acquiring the infection [59]. Universal childhood vaccination programmes were also introduced in some EU and EEA countries [60,61,62]. The rationale for the introduction of an extensive immunization offer was based on the evidence of the small effect and low coverage of selective vaccination strategies on reductions in disease incidence and of the high risk for large outbreaks [63].

Although childhood vaccination and improvements in the sanitary status have reduced the incidence of HAV infection in both developing and developed countries, HAV continues to be a leading reason for enterically spreading hepatitis worldwide, causing epidemics in both developing and industrialized nations [64,65]. Furthermore, changes in the socioeconomic conditions and the control mechanisms of the virus have triggered new circulation and transmission scenarios, with the subsequent need to study the virus in environmental and food matrices and the emergence of new susceptible populations (after the implementation of mass vaccination) [66].

In order to guide the choice of national vaccination strategy, the WHO recommends that countries collect and review data on the national burden of hepatitis A through surveys that estimate the age-specific prevalence of anti-HAV IgG antibodies, data on hepatitis A incidence, associated morbidity (hospitalization, fulminant hepatic failure, or liver transplantation), and mortality. Of note, in low- and very-low-endemicity settings, targeted vaccination should be considered [59].

Food handlers could be considered within the targeted HAV immunization strategies, since they have been found to be the most common source of published foodborne outbreaks [26,40,67]. Furthermore, HAV cases among this occupational category may be underestimated since, currently, there are no official archives of outbreaks involving food handlers [68]. HAV infections are reported at the community level, and reporting is not mandatory outside the local public health system. A review of the reported cases of hepatitis A due to food contamination could help to define the extent of the spread of the infection by food handlers.

The present study aims to summarize recent data on HAV vaccine performances and evidence from the published literature on recent foodborne HAV outbreaks and the role played by food handlers in HAV transmission. This could support policymakers and food business operators through the implementation of primary prevention strategies addressing HAV vaccines for food handlers.

## 2. The Virus, Its Clinical Outcomes, Diagnosis, and Treatment

Hepatitis A virus is an RNA virus classified in the genus *Hepatovirus* of the *Picornaviridae* family [69]. HAV has been enigmatic, eluding detailed structural analysis, for many years. Following the discovery of the virus particle in human faecal material via immunoelectron microscopy in 1973, there has been considerable controversy regarding the nature of the HAV genome and the nature of the virus [70]. It is an unusual hepatotropic virus with a long evolutionary history and multiple structural features that distinguish it from other mammalian picornaviruses, not only in terms of the nucleotide sequence of the genome but also in the structure of the capsid, which shares characteristics in common with primitive virus insects [8,69].

The HAV genome is a single-stranded, positive-sense RNA molecule, approximately 7500 nucleotides long, which directly serves as messenger RNA for the translation of proteins encoded by HAV [69,71]. It has an exceptionally low Guanine + Cytosine content compared to other picornaviruses. There is no cap at its 5′ end, which terminates instead in a covalent linkage to a small virally encoded protein, VPg, which is the protein primer for RNA synthesis, and, like cellular messenger RNAs, the 3′ terminates in a relatively long poly-Adenine tail [11,71,72]. As in other picornaviruses, the large open reading frame present in the genome of HAV may be divided into three functional regions, termed P1, P2, and P3. The P1 genomic region encodes the capsid polypeptides (VP1, VP2, VP3, and a putative VP4), and the P2 and P3 regions encode the non-structural polypeptides necessary for virus replication [73]. Nucleotide sequencing of the regions of the genome coding for the terminals of VP1, VP3, and for the VP1/2A junction region of HAV strains has demonstrated substantial heterogeneity of different HAV genotypes. Currently, seven main genotypes of HAV have been identified through nucleotide sequencing analysis: four humans (I, II, III, and VII) and three primates (IV, V, and VI). Within genotypes I and III, differences in nucleotides of about 7.5% allow one to define of sub-genotypes (IA, IB, IIIA, and IIIB) [10]. However, the antigenic structure of the capsid proteins is well preserved, and, consequently, there is only one human serotype HAV [69,74].

In contrast to many other picornaviruses, HAV does not disrupt cellular protein synthesis in infected cells and generally replicates without cytopathic effect. One of the most interesting features of the virus is its recently recognized ability for non-lytic release from infected cells as membrane-enveloped and quasi-enveloped infectious virions (eHAVs) [11,75]. There are two types of infectious viruses: naked, unenveloped HAV virions, which are released from exosomes via the action of bile salts in the passage to the gut and are shed in faeces, and quasi-enveloped virions (eHAV), created by taking over the host, which are contained in exosome-like vesicles and found in blood. The eHAV particles have a specific infectivity, similar to that of the naked virion, but the final infection process is still unknown [8,75,76].

HAV has anomalous physicochemical properties, which make it remarkably stable and resistant to temperatures up to 80 °C and pH up to 2 [8]. Disinfection is, therefore, complicated, especially the coating removal. HAV inactivation requires heating foods (>85 °C) for 1 min or disinfecting surfaces with a 1:100 dilution of sodium hypochlorite for 1 min [51,77].

After ingestion of HAV through the faecal–oral route, HAV survives the acidic stomach environment and is ultimately delivered to the liver, where it replicates.

Before the clinical manifestations of HAV infection, there are waves of viremia and copious amounts of viral shedding via faeces, which is the main source of HAV transmission. Therefore, the risk of transmission is highest during the prodromal phase before symptoms or biochemical manifestations [78].

Following an incubation period of 15–50 days (mean, 28 days) after HAV infection, patients can develop symptoms. The clinical manifestations of HAV infection range from asymptomatic infection to acute liver failure (ALF) but do not progress to chronic hepatitis [78,79]. The development of symptomatic hepatitis is associated with the patient’s age. Manifestation of symptoms is less common in children, while most adults (>70%) develop symptoms that persist for up to 8 weeks [79,80]. The onset of hepatitis A is often sudden, with fever (18–75%), malaise (52–91%), nausea or vomiting (26–87%), abdominal discomfort (37–65%), then dark urine (28–94%) and jaundice. Less commonly, itching, diarrhoea, arthralgia, or a rash develop. On physical examination, hepatomegaly (78%) and jaundice (40–80%) are frequently detected. Laboratory results show elevated levels of total bilirubin, alkaline phosphatase, and Alanine Transaminase (ALT) [78].

However, symptoms and biochemical laboratory findings are not specific for HAV infection; therefore, serological confirmation is essential for the diagnosis. Definitive diagnosis of hepatitis A infection is typically made through detection of serum immunoglobulin M (IgM) anti-HAV antibodies via enzyme immunoassay [81]. However, there is growing interest in faster diagnostic tests that could potentially facilitate the identification of susceptible individuals during outbreaks. Some immunochromatographic and salivary rapid tests have shown good sensitivity and good specificity for the detection of HAV immunoglobulins, with performances comparable to more traditional serological tests [82,83]. IgM antibodies typically peak about a month after exposure and can persist for up to a year. False-negative results can be seen in early infection, while the patient is viraemic, and IgM antibodies can be also detected in the setting of recent HAV vaccine boosting [84]. IgG response typically follows IgM response after 1 week, typically persists for life, and confers neutralizing activity to future HAV exposures [84,85].

Less than 5% of hepatitis A cases evolve into acute liver failure and require immediate transplant surgery [86]. Generally, supportive care, such as adequate hydration and symptomatic control of fever or vomiting, with antipyretics or antiemetics is generally performed [78,81]. Currently, there are no approved antiviral therapies for HAV infection. However, direct-acting antivirals have been evaluated in cell culture systems and shown to have potential effectiveness in inhibiting HAV replication and in antiviral activity [84,87]. Drug development and clinical trials are limited by the difficulty in enrolling subjects before they resolve their infection to measure potential outcomes of intervention [87].

## 3. Routes of Transmission and Epidemiology: A Focus on Food and Food Handlers

Hepatitis A virus (HAV) is primarily transmitted via the faecal–oral route, either through close person-to-person contact (e.g., household and sexual contacts of people infected with HAV) or consumption of contaminated food or water [21,47,88].

Additionally, sexual and parenteral transmission among men who have sex with men (MSM) and through infected syringes or blood components have been documented, respectively [89,90,91]. In detail, outbreaks involving MSM have been increasingly reported across Europe, North America, and Japan [59].

The global burden of HAV, estimated in 159 million acute infections in 2019, resulting in 39,000 deaths and 2.3 million disability-adjusted life years, is not uniformly distributed worldwide [48,49,92]. For example, 66% of acute hepatitis A cases and 97% of hepatitis A deaths occurred in low- and lower-middle-income countries [59]. The HAV epidemiology changes according to socioeconomic development and the subsequent improvement in hygienic and sanitary conditions [52,63,93]. Additionally, the incidence of HAV disease cases has declined substantially in countries with effective programs of immunization [52,94].

Considering the serological prevalence of IgG anti-HAV antibodies, more than 90% of subjects by the age of 10 years are seroprotected in most low-income regions, including Sub-Saharan Africa and parts of South Asia [95]. This is the result of frequent asymptomatic infections in early childhood and consequent induction of life-long protection [59]. Outbreaks are rare in these regions [59]. Nevertheless, almost all low-income countries contain an urban middle-class subpopulation composed of adolescents and adults who have neither been HAV-infected as children nor vaccinated and are, therefore, at high risk of serious HAV disease later in life [59].

In most high-income regions, there has been little circulation of HAV as a result of increased living standards; thus, a high proportion of individuals are susceptible among adults, and <50% of individuals are immune by the age of 30 years [95,96,97]. Of note, HAV infection in adolescents and adults is associated with a higher rate of severe clinical manifestations. Once the virus is introduced to these countries, either through contaminated food or person-to-person contact, outbreaks with an extensive spread of HAV and a subsequent heavy burden may be triggered [98]. Relevant social changes, such as the increased homeless population and drug use, in high-income countries have introduced low-income epidemiologic features within high-income countries [99,100]. In detail, several hepatitis A outbreaks have been reported in the U.S.A. since 2016, and available surveillance data indicate increased severity and higher mortality among the homeless population [59,99].

In middle-income regions of Asia, Latin America, Eastern Europe, and the Middle East, the available evidence regarding seroprevalence shows a mix of intermediate and low prevalence patterns [95]. In these regions, a significant proportion of adolescents and adults are susceptible, and regular community-wide HAV outbreaks occur. Thus, paradoxically, the transition from high to intermediate endemicity and a higher average age at infection are associated with an increase in the incidence of symptomatic hepatitis A cases [59].

Furthermore, improved sanitation and an increasing middle-income class in low- and middle-income countries have increased susceptible populations [101,102].

Of relevance, unequal distribution of wealth, large-scale migration, globalization of food, and urbanization/population density are resulting in changes in the epidemiological figures and in a new surge of HAV infections [101].

Food represents a relevant vehicle for HAV transmission to humans, and closed setting environments, restaurants, and catering services have been identified as common places of HAV outbreaks [21,103]. As a consequence of the industrialization of food production and globalization, many high-risk food items, such as shellfish, fresh or frozen fruits, and vegetables, are produced in HAV-endemic countries and consumed in developed countries [104,105], contributing to the HAV outbreaks in food-importing countries, where a substantial proportion of adults are susceptible [25,96,97,106,107,108,109,110,111].

However, the link between the contamination source and outbreaks could be untimely because foods can originate from distant locations, a single food can be used as ingredients in a wide variety of food items, and contamination titres are generally low [21]. Furthermore, foodborne HAV outbreaks may present as sporadic cases instead of outbreaks because of the long-lasting incubation period of the infection, the low viral counts in food, the difficulty for patients to remember food consumption history, and the possible missed reports of asymptomatic cases, which can still cause infection, making epidemiologic and laboratory investigations challenging [98,112]. Despite the fact that HAV cannot replicate in host-free environments, such as food and water, the low infective viral dose places consumers at high risk, irrespective of the level of contamination [21].

Furthermore, the persistence and stability of HAV in water, soil, environmental fomites, contaminated hands, and during transit through the stomach to the gut under different physical, chemical, and biological conditions are linked to the occurrence of relevant foodborne outbreaks [8,21,33,113,114].

As regards physical conditions, temperature is critical in terms of HAV persistence. The lower the temperature, the higher the persistence of HAV in several matrices (e.g., water, lettuce, carrots. fennel, green onions, spinach, stored leafy green, berry fruits, parsley and basil, mussels, oysters, and clams) [27,115,116,117,118,119,120,121,122,123,124]. Boiling or cooking food or liquids for at least 1 min at as high a temperature as 185 °F (85 °C) is required to inactivate the virus [125,126,127].

The persistence of HAV was also demonstrated under low-humidity conditions in different food matrices, such as lettuce, bell peppers, cantaloupe, and dried tomatoes [124,128,129,130,131].

Finally, HAV is resistant to low pH, as it needs to be able to withstand the acidity of the stomach in order to reach the lower intestinal tract in an infectious state [132,133,134]. Given its high resistance, HAV could survive in an infectious state from harvesting/processing to consumption, and faecal contamination of food with HAV can occur at any point in the food chain “from farm to fork” [21,33]. In particular, the most common routes of food contamination are contact with infected food handlers, with incorrectly treated sewage or sewage-polluted water, and, to a lesser extent, with contaminated surfaces [21].

Food handlers play a major role in the preparation of safe food, and they can spread the HAV infection to susceptible individuals who consume their prepared food, if infected themselves [40,67,68]. A single infected food handler can transmit the virus to dozens or hundreds of customers during food preparation or serving and cause a substantial economic burden to public health [26,135,136,137]. Indeed, food handlers have been recognized as a major source of many reported foodborne outbreaks worldwide [26]. Table 1 summarizes the main food-handler-associated incidents that are characterized by the identification of a food handler who worked during the potentially infectious period.

As highlighted in previous studies, HAV-infected food handlers present at the point of sale or who prepare food (such as in a restaurant, grocery stores, food market, and cafeterias) have been identified as the main sources of reported foodborne hepatitis A outbreaks [26]. Of relevance, food handlers employed in hospital food services were also identified as index cases.

Indeed, poor hand hygiene is a risk factor for viral contamination of foods or surfaces [142]. Microscopic quantities of faeces on hands could harbour sufficient virus particles to constitute a hazard since one milligram of faeces may contain up to 10^7^ genome copies in an immunocompetent HAV-infected person [143]. Furthermore, HAV is likely to be able to survive in an infectious state for at least several hours on hands [142,144].

In turn, contaminated surfaces can contribute to the transmission of HAV to hands or to food. HAV has been found in latrines and on toilet door handles in leafy green vegetable production sites [33,142]. The virus seems to be resistant to desiccation [145,146] and may persist in dried faecal material on a surface for several days. Refrigerated temperature and humidity are other relevant factors involved in the persistence of HAV on surfaces [114,146,147,148,149,150]. Hence, adequate sanitation and personal hygiene are important measures to control HAV transmission [16].

Furthermore, food contamination of fresh produce during primary production can occur through the water cycle via the use of sewage-polluted waters, used for irrigation in agriculture or for food washing, and also through sewage treatment plant by-products as fertilisers, such as sludge [33,115,148,151,152,153]. Viral particles could persist in an infectious state for several weeks in environmental waters, in soil, or organic waste after a contamination event, at least when ambient temperatures remain below 25 °C [115,148,153,154,155].

As regards HAV detection in food, this has been the most extensively studied form since the 1990s [16]. Any food matrices can be implicated in outbreaks because of potential cross-contamination, but the most common reported categories are ready-to-eat foods that are eaten raw and do not undergo further processing (e.g., washing/decontamination procedures), shellfish and bivalve molluscs, fresh leafy greens, and fresh and frozen berries, especially berries [21,25,107,108,110,111,128,129,156,157,158,159]. Despite variations among countries and in time, a high prevalence of HAV positive bivalve molluscan shellfish has been reported all over European countries (e.g., 4% HAV prevalence in Greece, 3–75% in Spain, 7.5% in Poland, and 0.9–23.2% in Italy) [21,30,160,161,162,163,164,165,166], in the United States (4.4%) [167], in China (5%) [168], in Thailand (3.8%) [169], in Japan (1.8%) [170], in Mexico (23.3%) [171], in Turkey (3.3%) [172], in Morocco (2.6%) [173], in Tunisia (26%) [174], and in Vietnam (1.7%) [175]. The consumption of raw or under-cooked shellfish, usually harvested from waters affected by the discharge of treated and untreated sewage, has been recognized as the most common causes of the abovementioned outbreaks [123,138,176].

Frozen or dried produce items, such as berry fruits and leafy green vegetables, that do not involve heating before consumption has been the vehicle of transmission of infection by HAV in several reported outbreaks, often involving large numbers of affected persons [25,26,27,28,33,109,110,128,129]. A prevalence of 25.8% was reported in fresh produce tested in Egypt [177], 2.8% in various frozen berries, frozen diced potatoes and frozen diced apple were tested in China [178], 0.001% in strawberries tested in South Korea [179], 1.9% in ready-to-eat salads in Italy [31], and <2% in berries and leafy greens tested at retail in Australia [180].

Fresh produce, such as leafy green vegetables and berry fruit, undergoes multiple manipulation processes, which increase the risk of their cross-contamination via direct contact with faecally contaminated hands of infected persons or contaminated surfaces [33]. In particular, strawberries and raspberries are often picked by hand, thus representing a high risk of viral contamination [33]. Furthermore, agricultural products grow close to the ground, which raises their contacts with sewage-polluted irrigation water or contaminated biosolids [21].

The abovementioned Table 1 also summarizes the main outbreaks involving food identified in the published literature, according to the study design, the date of the event, the country where they occurred, the implicated food, and the number of people involved. Despite most foodborne outbreaks linked to HAV being small, widespread outbreaks have been also observed and, consequently, multiple costly public health interventions are needed. Furthermore, a variety of foods were involved, and the sources of infection were identified in workers employed in restaurants and points of sale.

Since their first detection, HAV outbreaks due to food consumption have been documented mainly in the U.S.A. [16]. Then, the number of foodborne hepatitis A outbreaks associated with infected travellers and imported frozen produce items from endemic regions to developed countries such as Europe increased [112,181]. Europe has become the region with the highest number of registered foodborne outbreaks. A recently published systematic review by Andani A. et al. analysed HAV outbreaks occurring in Europe in the past two decades. Selected reports from 11 European countries were 118, the consumption of contaminated food was found among the main transmission routes, and the greatest number of foodborne outbreaks occurred recently, in 2017 [132]. Previously, between 2013 and 2014, Europe experienced another large and prolonged foodborne multistate HAV outbreak associated with the consumption of frozen berries, accounting to more than 1589 cases and two deaths [21,108,182].

Transnational outbreaks of foodborne infections are reported with an increasing frequency as a result of globalization and international food trade [98]. In the U.S.A., as the proportion of food imported from other countries increased (from 12% in 1990 to 20% in 2013), the number of foodborne outbreaks rose steadily [183,184]. As recently highlighted by Hu X. et al., the increase in imported foods from endemic regions through the global supply chain should make local and international health authorities, the food industry, and consumers of HAV contamination aware [98].

## 4. HAV Prevention and Current Recommendations on HAV Prevention Addressed to Food Handlers

No specific treatment is currently available for hepatitis A; therefore, prevention is extremely important. A prevention strategy is based on both pharmaceutical (i.e., immunoglobulins, vaccines) and non-pharmaceutical interventions (i.e., improving sanitary and hygienic conditions) so as to limit the circulation of the virus and its transmission throughout the community.

In terms of non-pharmaceutical interventions, an adequate sewage network and an effective waste collection and treatment system avoids the contamination of groundwater and represents a very effective general hepatitis A prevention strategy in industrialized countries [17]. Furthermore, water quality should be monitored regularly to ensure the provision of clean and safe drinking water for food handling and preparations [4].

Appropriate and regular hand hygiene practices, personally and during food preparation, are an effective measure to prevent the contamination of food and spread to other persons [185,186]. Although no specific food handler hygiene practice has been shown to reduce the likelihood of transmission, hygiene training for food handlers should include practical advice about the techniques of hand washing [144]. Furthermore, employers should provide access to hand-washing stations and sanitary facilities for field workers [26].

Reducing bare hand contact with foods that are not later cooked is a further preventative measure, and discouraging the presence of children in areas where food is harvested reduces the potential for the contamination of food during harvesting or processing [26].

In order to estimate whether a food handler has had recent infection and is potentially still capable of transmission, asymptomatic food handlers could be investigated for IgM anti-HAV and ALT levels. The results, in combination with likely dates of exposure, might be used to estimate whether the food handler has had recent infection and is potentially still capable of transmission. However, the validity of this approach is under evaluation. Anyway, employers and trainers for food handlers should stress the infectious risk and encourage ill food handlers to seek medical attention and to avoid duties that involve contact with food for at least 1–2 weeks after the onset of jaundice or until symptoms resolve [26].

Given the scientific evidence about the role played by food handlers in ensuring food safety standards, including those aimed at preventing the infectious risk, many international and national authorities provide and periodically update laws, recommendations, and guidelines [185,187,188,189,190,191,192,193,194,195,196,197,198,199,200,201,202,203,204,205,206,207,208,209,210,211,212,213,214,215,216,217,218,219,220,221,222,223,224,225,226,227] (Table S1 in Supplementary Materials). Thus, in accordance with the Food and Agriculture Organization of the United Nations (FAO), the World Health Organization (WHO) recommendations, the disposals of the main institutional authorities of developed countries (i.e., the European Commission, the U.S. Food & Drug Administration (FDA), the Canadian Department of Justice, the Food Standards Australia New Zealand (FSANZ) Board, the British Secretary of State, Food Standards Agency, and National Environment Agency, and the Food and Environmental Hygiene Department of Japan) and the guidelines developed by category associations or academic authorities, reducing HAV contamination of foods should be possible:

- Using approaches, such as Hazard Analysis and Critical Control Point (HACCP) systems by defining and monitoring specific critical points for hepatitis A contamination;
- Developing, implementing, and maintaining Good Hygiene Practices (GHPs) that support the production of safe and suitable food at all stages of the food chain, from primary production through to handling of the final product, assisting in controlling hazards in food products;
- Cultivating a positive food safety culture, acknowledging the importance of human behaviour in providing safe and suitable food among the personnel;
- Establishing effective systems that ensure cleanliness and, when necessary, adequate disinfection;
- Managing waste in food handling, food storage, and other working areas;
- Ensuring that those who come directly or indirectly into contact with food maintain appropriate personal health, maintain an appropriate degree of personal cleanliness, and behave and operate in an appropriate manner;
- Avoiding that personnel known or suspected to be ill or carrying a disease likely to be transmitted through food should access any food-handling area if there is a likelihood of them contaminating food and immediately reporting illness or symptoms to management.

The abovementioned general principles should be followed by food business operators (FBOs) and their personnel at all stages of the food chain, from primary production to distribution, consistent with the “farm to fork” strategy [228]. Thus, taking into account the stage in the food chain, the nature of the treated product, and the possible identified contaminants, these principles will enable FBOs to develop their own food hygiene practices and necessary food safety control measures, while complying with requirements set by the competent authorities that are responsible for overseeing food safety and suitability.

Guidelines on food safety management in a specific phase of the food chain (e.g., primary production, retail, catering) or information guides for food handlers on prevention of hepatitis A have been developed by category associations or academic authorities to further support FBOs in defining the self-control system of the food company for which they are responsible.

### 4.1. HAV Primary Prevention and Post-Exposure Prophylaxis

Prevention for Hepatitis A also includes passive post-exposure immunization with standard gamma globulin (antibodies), effective in producing short-term immunity of about three months. Immunoglobulins are preferred when rapid immunization is required [229]. Safe and effective vaccines, all based on the single serotype of the HAV, have been available since the early 1990s, making vaccination the key component of any prevention strategy [17]. Initially developed for individual prophylaxis, HAV vaccines are now increasingly used to control hepatitis A in endemic areas, in the hope of eliminating it in the long run [230]. Vaccines are effective for both pre-exposure and post-exposure prophylaxis and are gradually replacing passive prophylaxis [185,231]. Vaccines have several advantages over immunoglobulins: long-term immunity, low cost, ease of administration, and immediate availability.

HAV vaccination induces immune protection through both cellular and humoral pathways. The minimal protective level of anti-HAV IgG (correlated of protection) is still not universally defined: anti-HAV IgG antibody concentrations of ≥10 mIU/mL are considered seroprotective, although ≥20 mIU/mL is the most widely used cut-off in clinical trials [57]. The antibodies produced during the first few weeks include a low level of anti-HAV IgM antibodies, which, however, contribute significantly to the early immune response and allow for the use of the vaccine also for post-exposure prophylaxis [232]. Similar to natural infection, vaccine-induced immunity comprises a cellular mediated immunity response that establishes immune memory [230]. Three decades after the introduction of the HAV vaccine, assessing long-term immune responses has become a major issue. The protection conferred by the HAV vaccine is expected to be long-lasting, and the antibody titre ≥20 mIU/mL should persist for more than 20 years [233]. HAV vaccines have been shown to have an excellent safety and tolerability profile. Most of the documented post-vaccination adverse events have been mild for both the available inactivated and live attenuated vaccine types [230,234].

All inactivated HAV vaccines are licensed worldwide in a two-dose intramuscular schedule with a flexible interval between the first dose and the second dose (6–18 months). It is administered starting from the first year of age and induces 95% seroconversion in both healthy children and young adults (<40–50 years of age) after the first dose and almost 100% after the second dose, with antibody responses up to 10,000 mIU/mL [233].

Live attenuated HAV vaccines were developed in China in the early 1990s and have only recently been licensed in other countries (Bangladesh, Guatemala, India, Philippines, Thailand) [235]. Live attenuated vaccines are administered subcutaneously in a single-dose schedule, and the starting age for vaccination is 18 months. Considering single-dose vaccination, a similar seroconversion rate has been reported between attenuated and inactivated vaccines. However, the antibody response after two doses of inactivated vaccine is much higher.

The World Health Organization guidelines for pre-exposure vaccination are based on local epidemiology. In countries where the virus is endemic, most adults are naturally immunised, so the use of the vaccine is limited. Otherwise, the WHO recommends universal vaccination for countries with intermediate infection rates and only for risk groups in countries with low and very low infection rates. In some countries, such as Canada, Italy, Romania, and Russia, where the epidemiological pattern of hepatitis A is not homogeneous in all countries, local HAV childhood vaccination programs are recommended by the WHO [47,236]. In developed countries, where the population is mostly susceptible, transmission of the virus occurs mainly among high-risk groups. Current guidelines recommend HAV vaccination for specific groups of populations: people at increased risk of HAV infections (international travellers, men who have sex with men, drug addicts, homeless people, people exposed to occupational hazards, including food handlers, day-care centre staff, and garbage and sewage workers) and people at increased risk of severe disease and fulminant hepatitis (people with chronic liver disease, patients with blood-clotting disorders receiving blood-derived products, or patients affected by HIV) [47,76,236,237,238,239,240].

The goal of eliminating viral hepatitis by 2030 was put forward by the WHO, and more efforts should be made to achieve this result [186]. There is significant evidence of mass anti-HAV antibody seroprevalence (95–100% of population) after the implementation of universal HAV vaccination programs in some epidemic or endemic settings, resulting in a rapid decline in the incidence of hepatitis A cases [54,55,57,58,125,241,242,243,244,245,246,247] (Table S2).

### 4.2. The Implementation of Current Immunization Strategies: Hepatitis a Vaccination for Food Handlers

HAV is a virus of concern for food-processing facilities as this virus can be spread through food and shared utensils and for which the vaccine is among the most efficacious prevention methods, together with cleaning and sanitizing, hand washing, changing gloves, and staying home when sick.

The development of sanitation systems and effective washing by the food industry, together with good food cooking (at high temperatures and for adequate periods of time), reduces HAV contamination in food and prevents disease transmission [16,248].

Of note, new eating habits, especially in developed countries, consisting of seafood, leafy green vegetables, frozen fruits, and ready-to-eat salads, leading to further spread of new undercooked or raw food, and usually without any further washing/decontamination procedures, have led to the need to implement the management of supervened food safety threats [249].

Public health authorities, such as the Food and Drug Administration and European Council, also support these practices through the establishment of food safety standards described in several regulations and guidelines (e.g., Good Manufacturing Practice regulations, the Hazard Analysis and Critical Control Points (HACCP) system, the Food Safety Modernization Act (FSMA), and the Regulation (EC) No. 852/2004 on the hygiene of foodstuffs) [250,251].

Nevertheless, the last report presented by the European Commission about the official controls on food safety carried out by EU countries in 2021 in accordance with the Regulation (EU) 2017/625 analysed the most serious incidents of non-compliance, finding that the highest number of administrative sanctions and judicial actions was applied to the food and food safety area, including the adoption of hygienic measures [252]. Thus, a comprehensive approach to HAV prevention that includes primary prevention may be preferable.

This is of particular interest in the case of HAV because it is one of the vaccine-preventable foodborne diseases.

Furthermore, the high proportion of persons employed in the food industry makes food handlers a relevant target when hepatitis A outbreaks occur. Despite the impact of COVID-19 on employment, as of May 2023, there were 12,287.2 thousand employees in the United States [253], and 15.9 million people aged over 15 were employed in the food supply sector in the European Union (EU) in 2019 [254]. In a developed country such as the U.S.A., 8% of hepatitis A cases occurred in adults during the period of 1992–2000, and the occupational data reported were food handlers [26,255].

The recent “Guidance on reducing the risk of communicable disease transmission in food processing facilities” published by British Columbia–Ministry of Health includes HAV infection among illnesses that may be acquired in food facility work environments and vaccination against Hepatitis A among the prevention tools to limit illness transmission between workers [205].

In Europe, Austria and Germany have included food handlers as a target for HAV vaccines in their national immunization plans [256,257]. In late 1999, the vaccination of food handlers was also introduced as mandatory in St. Louis County, where sequential outbreaks of hepatitis A linked to food service handlers caused a huge strain on local health resources [258].

HAV vaccination of food handlers can be considered to be a prevention tool, useful both to manage the biologic risk to which this type of worker is exposed and to prevent an infectious hazard in agreement with the HACCP system. Regulation (EC) No 852/2004 of the European Parliament and of the Council of 29 April 2004, in fact, lays down general rules for food business operators on the hygiene of foodstuffs, taking account of the general implementation of procedures based on the HACCP principles that consist of identifying any hazards that must be prevented, eliminated, or reduced to acceptable levels [251].

Despite the current regulations on food hygiene, such as the Regulation (EC) No 852/2004, the “Food Code–2022 Recommendations of the United States Public Health Service Food and Drug Administration” [259], the Food Safety Standards in force in Australia and New Zealand [260], and the Handbook for Food Business about Safe Food for Canadians Regulations by the Canadian Food Inspection Agency [261], which enforce that no person suffering from or being a carrier of a disease likely to be transmitted through food is to be permitted to handle food or enter any food-handling area in any capacity if there is any likelihood of direct or indirect contamination [251], previous U.S. estimates have suggested that approximately 60% of food handlers with acute hepatitis A have worked during a time when they were potentially infectious, thus becoming a source for secondary transmission to their patrons [2].

Nevertheless, the estimated risk for secondary infection from hepatitis-A-infected food handlers to food establishment patrons in recent person-to-person outbreaks occurring in the U.S. was low (<1.0%), and pre-emptive food handler HAV vaccination is not seen as a cost-effective national policy to prevent large-scale transmission and foodborne outbreaks [240,262]. However, it must be noted that the cost of controlling outbreaks by providing post-exposure prophylaxis via immunization or immunoglobulin is also relevant [2].

Morey et al. estimated the financial burden of each public health response to hepatitis A outbreaks related to food handlers from data collected in 2012–2014 as more than USD 40,000 [67]. Indeed, evaluations, to be exhaustive, should balance the costs deriving from both the pre- and post-exposure vaccination.

In addition, favourable cost-effectiveness evaluations of vaccinating food service workers against hepatitis A infection estimated that vaccination of 100,000 food service workers would cost USD 8.1 million but reduce the costs of hepatitis A treatment, public health intervention, and work loss by USD 3.0 million, USD 2.3 million, and USD 3.1 million, respectively. From the societal perspective, a vaccination policy would reduce morbidity and mortality, while providing savings of USD 0.4 million [263].

A recent cross-sectional survey by Faour-Klingbeil D et al. aimed to explore the public perception of food safety after the COVID-19 pandemic and highlighted the increased awareness of food safety risks during the pandemic, even if there is no evidence that food transmits COVID-19. Furthermore, they found that vaccination affects how people perceive food safety and their food decision and that people seemed to be more reassured when knowing that workers in restaurants were vaccinated [264]. Even if HAV has a considerably lower epidemiological impact than COVID-19, the abovementioned observations can hypothetically be translated to HAV infection, which is a vaccine-preventable disease like COVID-19 but for which transmission via contaminated food or water has been strongly demonstrated. Furthermore, the increased attention for food safety demonstrated by consumers could be exploited by the restaurant industry, advertising a fully vaccinated workforce to attract customers to trust and eat at their restaurants.

Of note, food handlers are adult subjects, who are at higher risk of developing symptomatic and serious HAV disease [17,21,39].

In addition, food handlers could have some of the risk factors, such as active drug use and MSM, that make these individuals the main targets of hepatitis A vaccination and may also make them more difficult to reach, obtain consent for vaccination, and vaccinate [59]. In these cases, occupational immunization is a relevant opportunity to reach the target population. In countries such as the U.S.A. where the hepatitis A vaccine is routinely recommended [265], the vaccination of food handlers could be an opportunity to immunize those who have missed the vaccination.

Finally, vaccine costs are relatively low [266] and, thus, could be affordable for food business operators, and public health authorities could consider co-payment offers to encourage compliance. Food businesses may also consider potential economic savings deriving from avoiding worker absenteeism, post-exposure prophylaxis, and illness of customers.

Future evaluations, such as systematic reviews and meta-analyses, of the fragmented available evidence about foods contaminated by HAV and the ascertainment of food handlers as a source of infection could improve the awareness of the true scale of the problem and further support the immunization strategy addressed to food handlers.

## 5. Conclusions

In conclusion, an efficient hepatitis A primary prevention strategy to adopt in combination with the immunization of high risk groups may be to incentivize employers to vaccinate subjects who have or are seeking employment in the food industry, including those involved in processing and distribution phases. Public health departments may support the strategy by offering discounted vaccines. This could be seen as part of a comprehensive process towards reductions in the foodborne transmission of hepatitis A and the achievement of food safety.

## Figures and Tables

**Table 1:** Characteristics of selected published foodborne hepatitis A outbreaks and incidence associated with contaminated produce or infected food handlers.

Reference	Study Design	Years	Country	Identified Incidents/ Outbreaks	Implicated Food/ Index Case (Location)	**Cases (*n*.)**
[135]	Systematic review	1998–2004	Canada	16	Owner and chef, Bar worker, Food service worker, Part-time employee, Server (restaurant), Vegetable department worker, Food handler (food market), Food service worker, Food handler (hospital cafeteria, hospital food services), Food handler in produce department, Food service worker, Deli worker (grocery store), Worker (lounge)	47
[123]	Case-control study	1999–2008	Spain	2	Clams	284
[138]	Case-control study	2012–13	Netherlands	2	Mussels, raw onions, iceberg lettuce, leafy green lettuce	89
[139]	Case-control study	2019	England	1	School canteen food and bakery products	33
[140]	Case-control study	2017–18	Canada	1	Shrimp/prawns and blackberries	18
[98]	Review	2012–18	Developed countries ^1^	9	Frozen berries from Bulgaria and Poland, Pomegranate arils from Egypt and Turkey, Frozen cherries or pomegranate seeds from Egypt, Frozen bay scallops from Philippines, Frozen strawberries and blackberries from China, Frozen strawberries from Egypt and Morocco	2.369
[21]	Review	2009–19	Canada, United States, Australia, Hawaii, several European countries ^2^	6	Frozen pomegranate arils from Egypt and Turkey, Fresh dates from Iran, Sun dried tomatoes, Frozen mix berries from Bulgaria, Poland, Turkey, Frozen strawberries from Poland, Bakery products from Germany, Scallops from Philippines	2.534
[16]	Review	1956–2019	United States, Canada, England, Scotland, Ireland, China, Japan, several European countries, ^3^ South Korea, Hawaii, India, Philippines, Netherlands, Australia, New Zealand	119	Seafood (raw oysters, clams, mussels, shrimp), Vegetables, Frozen berries, Sandwiches, meat, cheese, drinks, ice cream, yogurt, salads, bakery products	323.391
[141]	Observational study	2019	United States ^4^	1	Fresh blackberries	20
[132]	Systematic review	1999–2021	France, Germany, Italy, Spain, United Kingdom	15	Liver pate, Shellfish (coquina clams, razor shells), Bakery products, Filled doughnuts, Butcher shop products, Frozen berries, Sandwiches, Water sources, Semi-dries tomatoes, School canteen food, Imported dates	4.593

^1^ Australia, Sweden, United States, New Zealand, Europe economic area countries, Denmark, Finland, Norway, Canada; ^2^ Austria, Sweden, Netherlands, France, Germany; ^3^ Italy, France, Belgium, Germany, Spain, Finland, Norway, Sweden, Austria; ^4^ Nebraska, Wisconsin, Indiana, Michigan, Minnesota, Missouri, Pennsylvania. Searching strategy: food handler-associated incidents and outbreaks involving food searched in PubMed since 1 January 2006 to 31 May 2023. The terms on hepatitis A, food, food handlers, incidence and outbreak have been combined. Only studies published in English were selected.

## Data Availability

No new data were created or analyzed in this study. Data sharing is not applicable to this article.

## Supplementary Materials

The following supporting information can be downloaded at: https://www.mdpi.com/article/10.3390/vaccines11071271/s1, Table S1: International and national laws, recommendations and guidelines on food safety specifically enforced for Food Business Operators (FBOs); Table S2: Inactivated and live attenuated vaccine performances evaluated by selected published reviews.
