# Supplementary material for: Hepatitis a Vaccine as Opportunity of Primary Prevention for Food Handlers: A Narrative Review
Source: Vaccines (Basel). 2023 Jul 21;11(7):1271. doi: 10.3390/vaccines11071271 (PMC10383099; doi:10.3390/vaccines11071271)
Supplement: Supplementary file 1 [file vaccines-11-01271-s001.zip › vaccines-2504838-supplementary.docx]

**Supplementary Material**

**Table S1.** International and national laws, recommendations and guidelines on food safety specifically enforced for Food Business Operators (FBOs)

| **Continent** | **Authority** | **Title** | **Ref.** |
| --- | --- | --- | --- |
| World | FAO and WHO | General Principles of Food Hygiene | [187] |
|  | FAO | Food Chain Crisis Management Framework – EMPRES Food Safety | [185] |
| Europe | EC | REGULATION (EC) No 852/2004 OF THE EUROPEAN PARLIAMENT AND OF THE COUNCIL on the hygiene of foodstuffs of 29 April 2004 | [188] |
|  |  | REGULATION (EC) No 183/2005 OF THE EUROPEAN PARLIAMENT AND OF THE COUNCIL of 12 January 2005 laying down requirements for feed hygiene | [189] |
|  |  | Commission Notice on the implementation of food safety management systems covering Good Hygiene Practices and procedures based on the HACCP principles, including the facilitation/flexibility of the implementation in certain food businesses (Official Journal of the European Union, C 355, 16 September 2022) | [190] |
|  |  | Commission Notice providing guidance on food safety management systems for food retail activities, including food donations (Official Journal of the European Union, C 199, 12 June 2020) | [191] |
|  |  | Commission notice on guidance document on addressing microbiological risks in fresh fruits and vegetables at primary production through good hygiene | [192] |
|  |  | Guidance document on the implementation of certain provisions of Regulation (EC) No 852/2004 On the hygiene of foodstuffs | [193] |
|  |  | Community Guide to the Principles of Good Practice for the Microbiological Classification and Monitoring of Bivalve Mollusc Production and Relaying Areas with regard to Implementing Regulation 2019/627 | [194] |
|  | World Union of Wholesale Markets – European Regional Section | Community guide to Good Hygienic Practices specific to wholesale market management in the European Union | [195] |
|  | European Federation of Bottled Waters | Guide to good hygiene practices for packaged water | [195] |
|  | Belgian Federal agency for food chain security | Self-control guide for Restaurants, Hotels & Pubs | [196] |
|  | Belgium, Federal agency for food chain security | Self-control guide for retailers | [196] |
|  | Italian category associations | GHPs manuals | [197] |
|  | Irish National disease surveillance centre | Preventing Foodborne Disease: A Focus on the Infected Food Handler | [198] |

**Table S1.** International and national laws, recommendations and guidelines on food safety specifically enforced for Food Business Operators (FBOs) (cont.)

| U.S.A. | FDA | Food code 2022 | [199] |
| --- | --- | --- | --- |
|  |  | Food Safety Modernization Act (FSMA) | [200] |
|  | North Dakota State University | Food safety Basics: a reference guide for foodservice operators | [201] |
|  | University of Washington | Hepatitis A Information for Food Establishment | [241] |
|  | Washington State Department of Health | Washington State Retail Food Code | [202] |
|  | N.C. Department of Health and Human Services | North Carolina Food Code Manual | [203] |
|  | British Columbia Centre for Disease Control | Food Premises Guidelines | [204] |
|  | British Columbia – Ministry of Health | Vaccination: Guidance on reducing the risk of communicable disease transmission in food processing facilities | [205] |
|  | FMI Food Protection Committee | Hepatitis A information guide for food retail | [206] |
|  |  | Hepatitis A information guide | [207] |
|  | Oklaoma State Department of Health | Hepatitis A and food handlers | [208] |
|  | New York State Department of Health | Hepatitis A and Food Service Workers | [209] |
|  | San Bernardino County, Public health Environmental health services | Hepatitis A and food handlers | [210] |
|  | City of Long Beach, Department of health and human services | Hepatitis A Prevention Guidelines for Food Handlers | [211] |
|  | Hawaii State Department of health | Hepatitis A Information for Food Service Establishments | [212] |
|  | Minnesota Department of Health | Food Employee Illness Guidelines | [213] |
|  | County of San Diego,  Department of Environmental Health and Quality -  Food and Housing Division | The Health Of The Public  Is In Your Hands. A Food Handler’s Guide  To Food Safety | [214] |

**Table S1.** International and national laws, recommendations and guidelines on food safety specifically enforced for Food Business Operators (FBOs) (cont.)

| Canada | Department of Justice | Food and Drugs Act | [215] |
| --- | --- | --- | --- |
|  |  | Food and Drug Regulations | [216] |
|  | Ontario, Ministry of Health and Long-term care | Food Safety: a guide for Ontario’s food handlers | [217] |
| Australia | Food Standards Australia New Zealand (FSANZ) Board | Food Standards Code | [218] |
|  |  | Safe Food Australia Guide | [219] |
|  | Victoria State Government | Food safety supervisors and training - Guide for food businesses | [220] |
|  | Queensland Government | Food Handler Exclusion Guidelines | [221] |
| UK | Secretary of State | The Food Safety and Hygiene (England) Regulations 2013 | [222] |
|  | Food Standards Agency | Food Standards Agency Food and Feed Law Guide | [223] |
|  |  | Food hygiene for businesses | [224] |
|  | National Environment Agency | Food handler’s handbook | [225] |
|  | Royal College of Physicians – NHS Plus | Infected food handlers – Occupational aspects of management | [226] |
| Japan | Food and Environmental Hygiene Department | Food Hygiene Code | [227] |

Food and Agriculture Organization of the United Nations, FAO; World Health Organization, WHO; EC, European Commission; FDA, U. S. Food and Drug Administration

**Table S2.** Inactivated and live attenuated vaccine performances evaluated by selected published reviews.

| **Ref.** | **Country** | **Age** | **Vaccine type**  **and schedule** | **Immunigenicity*** | **Other outcomes**** |
| --- | --- | --- | --- | --- | --- |
| [241] | U.S.A.  Argentina  China  Europe^1^ | Children  Adults | IV 2 doses  0,6 mo  0,6 mo | SR= 99.09% and GMC= 61.59-390 mIU/ml (FU 10 ys)  GMC= 684 mIU/ml (FU 6-10 ys)  SR= 100% and GMT= 522 mIU/ml (FU 5.5 ys) | 0 cases per 100.000 (FU 15 ys) |
|  |  |  | IV 2 doses  0,12 mo | GMC= 1321 mIU/ml (95% CI: 70-2482) (vaccine 1), 1208 mIU/ml (vaccine 2), 859 mIU/ml (95% CI: 457-1612) (type2/type 1), 1191 mIU/ml (FU 5-6 ys) (vaccine 1/vaccine 2)  SR= 99.3% and GMC= 749-1321 mIU/ml (FU 5-6 ys)  GMC= 526 mIU/ml (FU 9-11 ys)  GMC females= 741 mIU/ml and GMC males= 332 mIU/ml (FU 9-11 ys) |  |
|  |  |  | IV 2 doses  6,18 mo after HIV diagnosis | SR= 85% and GMC= 64 mIU/ml in HIV-positive individuals (FU 6-10 ys)  GMC= 70 in individuals with CD4 ≥350 cells/mm3: mIU/ml (FU 6-10 ys)  GMC= 50 mIU/ml in individuals with CD4 < 350 cells/mm3 (FU 6-10 ys) |  |
|  |  |  | IV 3 doses  0,1,2 mo  0,1,6 mo  0,1,12 mo | SR= 86% and GMC= 131 mIU/ml (FU 14 ys)  SR= 100% and GMC= 227 mIU/ml (FU 14 ys)  SR= 100% and GMC= 212 mIU/ml (FU 14 ys) |  |
|  |  |  | IV 4 doses  0,1,2,12 mo | GMT= 506 mIU/ml (Dos 180 El.U) (FU 6-6.3 ys)  GMT= 983 mIU/ml (Dos 360 El.U) (FU 6-6.3 ys)  GMT= 1587 mIU/ml (Dos 720 El. U) (FU 6-6.3 ys) |  |
|  | China | Children | LAV  1 dose | SR= 97.65% and GMC= 67.87 mIU/ml (FU 3.6 ys)  SR= 71-75% and GMC= 80-89 mIU/ml (FU 8 ys)  SR= 80.2% and GMT= 145 mIU/ml. (FU 10 ys)  SR= 81.3% and GMT= 128 mIU/ml (FU 15 ys) | N.A. |
|  |  |  | LAV  2 doses  0,6 mo | SR= 100% and GMC= 84.6-174.1 mIU/ml (FU 7 ys)  SR= 98% and GMC= 262.8 mIU/ml (FU 8 ys) |  |
|  |  |  | LAV 3 doses  0,6,12 mo  0,2,6 mo  month | SR= 100% and GMC= 336.8 mIU/ml (FU 7 ys)  SR= 100% and GMC= 480.6-918.2 mIU/ml (FU 8 ys) |  |

**Table S2**. Inactivated and live attenuated vaccine performances evaluated by selected published reviews (cont.)

| **Ref.** | **Country** | **Age** | **Vaccine type**  **and schedule** | **Immunigenicity*** | | **Other outcomes**** |
| --- | --- | --- | --- | --- | --- | --- |
| [58] | Any origin | Any age | IV 1 dose  2 doses  0,6 mo  3 doses  0,1,6,12 mo | SR= 99.45% (FU 7 and 17 mo) | 9/20,684 cases (0.04%) in VG, 92/20746 cases (0.44%) in CG  10/20,812 cases (0.05%) in VG, 95/20978 cases (0.45%) in CG  RRR: 0.89-0.90.  Complications and mortality reduction per 100000: 0.07% in HAV VG, 0.05% in CG.  AE   - Serious: not reported - Non-serious local: 11.41% in vaccine group and 9.76% in control group - Non-serious systemic: 6.28% in VG and 6.38% in CG | |
|  |  |  | LAV 1 dose | N.A. | 21/354914 cases (0.01%) in VG, 410/335776 cases (0.12%) in CG  RRR: 0.95 | |
| [242] | China | Children  >18 mo | IV 1 dose | SR= 63-100% and GMC= 146-382 mIU/ml (FU 1 mo) (vaccine 1) | N.A. | |
|  |  | Adults |  | SR= 50-94% and GMC= 139-175 mIU/ml (FU 1 mo) (vaccine 1) |  |  |
|  |  | Children  >18 mo | IV 2 doses  0,3 mo  0,6 mo | SR= 100% and GMC= 1973-21696 mIU/ml (FU 1 mo) (vaccine 1)  SR= 100% and GMC= 285-496 mIU/ml (FU 3 ys) (vaccine 1)  SR= 100% and GMC= 339 mIU/ml (FU 4 ys) (vaccine 1)  SR= 100% and GMC= 261 mIU/ml (FU 5 ys) (vaccine 1)  SR= 100% and GMC= 412 mIU/ml (FU 3 ys) (vaccine 2)  SR= 100% and GMC= 222 mIU/ml (FU 4 ys) (vaccine 2)  SR= 100% and GMC= 180 mIU/ml (FU 5 ys) (vaccine 2) |  |  |
|  |  | Adults |  | SR= 100% and GMC= 1066-2747 mIU/ml (vaccine 2) |  |  |
| [243] | China | >18 mo | IV 1 dose | SR= 62-100% and GMC= 28-3630 mIU/ml (FU 1 mo) | 0/108 cases in VG, 4/115 cases in CG (FU 2 mo, VE 100%)  0/3.365 cases in VG, 4/2.572 cases in CG (FU 4 mon, VE 100%)  AE:   - Serious: not reported - Fever (1-8%) | |
|  |  |  | IV 2 doses  0,1 mo  0,3 mo  0,6 mo | SR= 100% (FU 1 mo) |  |  |
|  |  |  | LAV 1 dose | SR= 74-100% and GMC= 41-1607mIU/ml (FU 2 mo);  GMC= 75-119 mIU/ml (FU 3 months)  SR= 56-100% and GMC= 42-1945 mIU/ml (FU 6 mo) | 0/260.117 cases in VG, 37/235.235 cases in CG (FU 0.5 ys, VE 100%)  5/208.328 cases in VG, 66/192.448 cases in CG (FU 1 yr, VE 93%)  1/21 case in VG, 119/237 cases in CG (FU 1 yr, VE 98%)  0/84.412 cases in VG, 22/79.254 cases in CG (FU 1 yr, VE 100%)  2/3.771 cases in VG, 71/3.545 cases in CG (FU 3 ys, VE 97%)  1/10.459 case in VG, 20/13.005 cases in CG, 1/1.804 case in VG and 14/1.653 cases in CG (FU 3.5 ys, VE 94%)  0/72.408 cases in VG, 495/242.168 cases in CG (FU 4 ys, VE 100%)  1/15.779 case in VG, 38/60.517 cases in CG (FU 5 ys, VE 90%)  0/745 cases in VG, 11/475 cases in CG (FU 10 ys, VE 100%)  AE:   - Serious: not reported - Fever (0.4-5%), - Rash (0-1.1%) - ALT increase (0.015%) | |
|  |  |  | LAV 2 doses  0,6 mo | SR= 100% and GMC= 218-1586 mIU/ml (FU 7 months-7 ys) |  |  |
|  |  |  | LAV 3 doses  0,6,12 mo | SR= 100% and GMC= 337-1945 mIU/ml (FU 13 months- 7 ys) |  |  |

**Table S2**. Inactivated and live attenuated vaccine performances evaluated by selected published reviews (cont.)

| **Ref.** | **Country** | **Age** | **Vaccine type and schedule** | **Immunigenicity*** | **Other outcomes**** |
| --- | --- | --- | --- | --- | --- |
| [244] | Canada  U.S.A.  Argentina  Brazil  Mexico  Bahamas  Europe^2^  South Africa  China  Australia | 1-18 ys | Combined HAV-HVB 3 doses  0,1,6 month | Vaccine 1  SR= 89.5-92.3 and GMC= 221-254 mIU/ml (FU 1 mo)  SR= 98.7-100.0 and GMC= 574-665 mIU/ml (FU 2 mo)  SR= 97.4-100.0 and GMC= 307-377 mIU/ml (FU 6 mo)  SR= 100.0 and GMC= 5694-6260 mIU/ml (FU 7 mo)  Vaccine 2  SR= 93.2% and GMC= 227 mIU/ml (FU 1 mo)  SR= 99.3% and GMC= 549 mIU/ml (FU 2 mo)  SR= 99.3% and GMC 299 mIU/ml (FU 6 mo)  SR= 100% and GMC 4174-9257 mIU/ml (FU 7 mo)  SR= 100% and GMC 233-761 mIU/ml (FU 10 ys) | From 5.44 to 3.02 per 100.000 (-45%) (Catalonia) (FU 14 ys)  AE:  Vaccine 1   - Serious: 0-9,5% - Non-serious local: Soreness/pain (10.5-65.6%), Redness (0.0-26.0%), Swelling (0-20.6%) - Non-serious systemic: Fever (0.5-7.8%), Headache (2.1-38.7%), Malaise (0.5-29.0%), Fatigue (2.6-42.2%), Nausea (1.3-11.9), Vomiting (0-7.0), Gastrointestinal (1.7-7.5%)   Vaccine 2   - Serious: 0,4-0,8% - Non-serious local: Soreness/pain (39.1-52.2%), Redness (11.9-30.8%), Swelling (4.9-21.1%), - Non-serious systemic: Fever (1.3-15.6%), Headache (13.9-42.6%), Fatigue (19.3-32.0%), Gastrointestinal (3.4-30.3%)   Vaccine 3   - Serious: 0-1,7% - Non-serious local: soreness/pain (13.8-54.0%), Redness (1.0-29.4%), Swelling (0.8-15.7%) - Non-serious systemic: Fever (1.7-13.3%), Headache (7.0-31.5%), Malaise (7.5%), Fatigue (5.0-29.2%), Nausea (4.4%), Vomiting (1.2%), Gastrointestinal (4.3-22.9%) |
|  |  |  | Combined HAV-HVB 3 doses  0,7,21 day | Vaccine 1  SR= 87% and GMC= 58 mIU/ml (FU 21 days)  SR= 100% and GMC= 424 mIU/ml (FU 2 mo)  SR= 100% and GMC= 1416 mIU/ml (FU 6 mo) |  |
|  |  |  | Combined HAV-HVB 2 doses  0,6 mo | Vaccine 2  SR= 97.8-100% and GMC=275-8203 mIU/ml  Vaccine 3  SR= 100% and GMC 50.6-80.7 mIU/ml (FU 10 ys)  GMC= 3701-14790.1 mIU/ml (FU 7 months) |  |
|  |  |  | Combined HAV-HVB 2 doses  0,12 mo | Vaccine 3  SR=99% and GMC= 293.9 mIU/ml (FU 1 month)  SR= 84.2% and GMC= 146.5 mIU/ml (FU 12 months)  SR= 99% and GMC= 8471.8 mIU/ml (FU 13 months)  SR= 98.8% and GMC= 1704.7 mIU/ml (FU 2 ys)  SR= 98.8% and GMC= 1273.6 mIU/ml (FU 3 ys)  SR= 100% and GMC= 880.6 mIU/ml (FU 5 ys)  SR= 100% and GMC= 932.6 mIU/ml (FU 6 ys) |  |
|  |  |  | Combined HAV-HVB 4 doses  0,7,21 day-12 mo | Vaccine 1  SR= 98,5-100% and GMC= 454.6-845 mIU/ml (FU 1 months)  SR= 100% and GMC= 480-628 mIU/ml (FU 3 months)  SR= 96.2-96.9 and GMC= 374 mIU/ml (FU 12 months)  SR= 100% and GMC= 7110.5-9571 mIU/ml (FU 13 months) |  |
|  |  | >18 yrs | Combined HAV-HVB 3 doses  0,1,6 mo | Vaccine 1  SR= 90-98 and GMC= 91.3-471 mIU/ml (FU 1 month)  SR= 29-100 and GMC= 17-1311 mIU/ml (FU 2 months)  SR= 71-100 and GMC= 173-557 mIU/ml (FU 6 months)  SR= 88-100 and GMC= 2455-8895 mIU/ml (FU 7 months)  SR=97.3-100% and GMC= 343.6-1824 (FU 2-15 ys) |  |

**Table S2**. Inactivated and live attenuated vaccine performances evaluated by selected published reviews (cont.)

| **Ref.** | **Country** | **Age** | **Vaccine type and schedule** | **Immunigenicity*** | **Other outcomes** |
| --- | --- | --- | --- | --- | --- |
| [245] | U.S.A.  Argentina  Panama  Uruguay  Israel  Europe^3^  China | >1 yrs | IV 1 dose  IV 2 doses  0,6-12 mo  IV 3 doses  0,1,2 mo  0,1,6 mo  0,1,12 mo | GMC= 18-591 mIU/ml (FU 5-17.3 ys | From 69.6 to 2.7 cases (-96%) per 100,000 (FU 5 ys)  From 2-3 to 2 cases (-20%) per 100.000 (FU 6 ys)  From 142.4 to 7.6 cases (-95%) per 100,000 (FU 9 ys)  From 51.1 to 3.7 cases (-93%) per 100.000 (FU 10 ys)  From 66.5 to 7.9 cases (-88.1%) per 100,000 (FU 11 ys)  From 6.0 to 0.4 cases (-93%) per 100.000 (FU 12 ys)  From 41 to 2.6 cases (-94%) per 100.000 (FU 13 ys)  From 22.2 to 0.9 cases (-95.9%) per 100,000 (FU 13 ys)  From 10.7 to 2.6 cases (-76%) per 100,000 (FU 13 ys)  From 50.4 to <1.0 cases (>-98%) per 100,000 (FU 19 ys)  Mortality rate per 100.000: from 0.1 to 0.02 (FU 12 ys) and from 0.51 to 0.28 (age adjusted) (FU 14 ys)  Hospitalizations per 100.000: from 0.64 to 0.29 (FU 7 ys) and from 7.3 to 24.5 (FU 12 ys).  Hospital admission per 1000 admission among children aged 0-14 ys: from 50.5 to 20.8 (FU 14 ys).  Outbreaks cases in day care and school settings per year: from 45.6 to 0 (FU 12 ys).  Fulminant hepatic failure cases per year: from 17.5 to 0 (FU 11 ys  Ambulatory visits per 100.000: from 20.9 to 8.7 (FU 8 ys). |
| [246] | U.S.A.  Europe^4^  Korea | Adults | IV 1 dose | SR= 90-94% and GMC= 106-169 mIU/ml in healthy subjects.  SR= 0-67% and GMC= 0-101 mIU/ml (FU 1-2 months) in organ transplant recipients using drugs for prevention of rejection:   - Liver transplant recipients: SR= 59% (FU 2 ys), SR= 8% (FU 1 months), SR=19% (FU 6 months); - Kidney transplant recipients: SR= 26% (FU 2 ys)   SR= 6-100% and GMC= 7.5-84 mIU/ml, SR= 10-68% (FU 1 mo),  SR= 33-87% (FU 2 mo) in patients with chronic inflammatory conditions using TNF-alpha/ conventional immunomodulators.  SR= 47% and GMC= 30 mIU/ml in patients using Rituximab. | N.A. |
|  |  | Adults | IV 2 doses  0,1 mo  0,2 mo  0,6-12 mo | SR= 100% and GMC= 920-1.592 mIU/ml in healthy subjects.  SR= 0-97% and GMC= 0-1.306 mIU/ml (FU 1-2 mo), SR= 26-59% (FU 2 ys) in organ transplant recipients using drugs for prevention of rejection.  SR= 48-100% and GMC= 109.3-432 mIU/ml, SR= 93% (FU 2 ys) in patients with chronic inflammatory conditions using TNF-alpha/ conventional immunomodulators.  SR= 61% in patients using Rituximab. | N.A. |

**Table S2**. Inactivated and live attenuated vaccine performances evaluated by selected published reviews (cont.)

| **Ref.** | **Country** | **Age** | **Vaccine type and schedule** | **Immunigenicity*** | **Other outcomes** |
| --- | --- | --- | --- | --- | --- |
| [125] | China  India | >1 yr | LAV 1 dose | SR= 98,6% and GMC= 287 mlU/ml (FU 2 mo)  SR= 93.6% and GMC= 226 mlU/ml (FU 1 yr)  SR= 83.3% and GMC= 173 mlU/ml (FU 6 ys)  SR= 80.2-98.1% and GMC= 145 mlU/ml (FU 10 ys)  SR= 81.3% and GMC= 128 mlU/ml (FU 15 ys)  SR= 62% and GMC 64.8 mlU/ml (FU 17 ys)  SR= 70.54% (age 3 ys)  SR= 61.82% (age 6 ys)  SR= 63.87% (age 9 ys)  SR= 65.83% (age15 ys)  SR= 71.78% (age18 ys)  SR= 77.14% (age 25 ys)  SR= 92.08% (age 35 ys) | From 1203.8 to 0 (-100%) per 1000.000 (FU 6 ys) |
|  |  |  | LAV 2 doses | GMC= 1832.1 mlU/ml (FU 2 wks) |  |
| [57] | U.S.A.  Argentina  Nicaragua  Europe^5^  India  China | Children | LAV  1 dose | SR= 62.0-98.0% and GMC= 64.8-262.8 mlU/ml (FU 5-17 ys) | N.A. |
|  |  |  | LAV 2 doses  0,1-5 yrs | SR= 84.6% and GMC 661.3 mIU/ml (FU 17 ys)  SR= 100% and GMC 3133.5 mIU/ml (FU 8 ys)  GMC= 955.1 mIU/ml (FU 5 ys) |  |
|  |  |  | IV 1 dose | SR= 85.9-100% and GMC= 76.3-122.5 mlU/ml (FU 4-10 ys) |  |
|  |  |  | IV 2 doses  0,6 mo  0,12 mo | SR= 30-100% and GMC= 11-601.6 mlU/ml (FU 10-20 ys) |  |
|  |  |  | IV 3 doses  0,1,2 mo  0,1,6 mo  0,1,12 mo | SR= 76.5-100% and GMC= 60-511.9 mIU/ml (FU 10-20 ys) |  |

**Table S2**. Inactivated and live attenuated vaccine performances evaluated by selected published reviews (cont.)

| **Ref.** | **Country** | **Age** | **Vaccine type and schedule** | **Immunigenicity*** | **Other outcomes** |
| --- | --- | --- | --- | --- | --- |
| [247] | U.S.A.  Uruguay  Panama  Nicaragua  Greece  Israel  Kingdom of Saudi Arabia  China | ≥ 12 mo | IV 1 dose | SR≥ 90.0% and GMC= 21-712.5mIU/ml (FU 15 ys)  SR= 67.4% (95%C.I.:64.7%-70.0%) in Israelian Jewish and 88.2% (95%C.I.:86.1%-90.2%) in Arab  SR= 74.3% and GMC 40.2 mIU/ml (FU 8.1 ys)  SR= 96.7% and GMCs= 125.6mIU/ml) (FU 7 ys) | From 4.57-142.4 to 0.4-20.7 cases (-98.1%) per 100.000 (FU 1-14 ys)  Hospitalization rate per 100.000: from 0.81-50.5 to 0.26-20.8 (-58.8-68.5%)  Ambulatory visits per 100.000: from 12.9 to 7.5 (-41.5%)  Complications and mortality reduction per 100.000: from 0.038 to 0.026 (-32%)  Infection incidence in vaccinated vs unvaccinated 1.5-18-year-olds = 22.9% at six weeks and 100% at 6 weeks-15 months post-vaccination  Infections in vaccinated children  and estimated infections  in hypothetically unvaccinated children 3-18-year-olds = 98.3% (FU 7.5 ys)  Infection incidence in 2-17-year-olds immunized with ≥ 1 dose vs unvaccinated children of same age = 98%  (FU 5 ys)  Infection incidence in 1-17-year-olds at the time of vaccination program initiation vs after 4 ys = 95% |
|  | Argentina  Brazil | 11mo- 18 yrs | IV, 1 dose | SR=74.3-100% and GMC=40.2-170.5 mIU/ml (FU 7-8.1 ys) | From 3.0-85.5 to 0.5-10.2 cases (-83.4-88.1% per 100.000 (FU 3-6 ys)  Complications and mortality reduction per 100.000: from 0.015-0.026 to 0.0009-0.014  Fulminant hepatic failures per yr: from 17-27 to 0-13  Liver transplants per yr: from 8-27 to 0-9 (FU 5 ys)  Infection incidence in vaccinated vs unvaccinated children 1.5-6-year-olds) = 22.9% (FU 6 weeks) and 100% (FU 6 weeks-15 months)  Infections in vaccinated  children vs estimated infections in hypothetically unvaccinated 3-18-year-olds = 98.3% (FU 7.5 ys) |

Abbreviations: IV, inactivated vaccine; LAV, live-attenuated vaccine; Age (mo), age at administration (months); SR, Antibody seropositivity rates; GMCs; FU (ys/mo), seroprotection duration (years/months); N.A., not available; wks, weeks; Relative risk reduction (RRR); AE, Adverse events; VG, vaccine group; CG, control group.

^1^Switzerland, Austria, Belgium; ^2^Austria, Switzerland, Spain, Germany, Norway, Sweden, Denmark, Belgium, Estonia, Lithuania, Czech Republic, Hungary, The Netherlands; ^3^Greece, Belgium; ^4^Germany, Netherlands, Sweden, Greece; ^5^Belgium, France, Switzerland.

*SR (%);GMCs (mIU/ml); ** Clinically confirmed hepatitis A cases (n. and incidence), RRR, Complications and mortality reduction per 100.000, AE (%), Hospitalizations per 100.000, Ambulatory visits per 100.000

Searching strategy: reviews and systematic reviews on HAV vaccine performances searched in Pubmed since January 1, 2012 to May 31, 2023. The terms on hepatitis A vaccine, immunogenicity, efficacy, effectiveness, tolerability, safety have been combined. Only studies published in English were selected
